# ThrRS-Mediated Translation Regulation of the RNA Polymerase III Subunit RPC10 Occurs through an Element with Similarity to Cognate tRNA ASL and Affects tRNA Levels

**DOI:** 10.3390/genes14020462

**Published:** 2023-02-10

**Authors:** Ofri Levi, Monalisha Mallik, Yoav S. Arava

**Affiliations:** Faculty of Biology, Technion—Israel Institute of Technology, Haifa 3200003, Israel

**Keywords:** aminoacyl tRNA synthetase, RNA binding proteins, ThrRS, tRNA, anticodon stem-loop, RNA polymerase III, RPC10

## Abstract

Aminoacyl tRNA synthetases (aaRSs) are a well-studied family of enzymes with a canonical role in charging tRNAs with a specific amino acid. These proteins appear to also have non-canonical roles, including post-transcriptional regulation of mRNA expression. Many aaRSs were found to bind mRNAs and regulate their translation into proteins. However, the mRNA targets, mechanism of interaction, and regulatory consequences of this binding are not fully resolved. Here, we focused on yeast cytosolic threonine tRNA synthetase (ThrRS) to decipher its impact on mRNA binding. Affinity purification of ThrRS with its associated mRNAs followed by transcriptome analysis revealed a preference for mRNAs encoding RNA polymerase subunits. An mRNA that was significantly bound compared to all others was the mRNA encoding RPC10, a small subunit of RNA polymerase III. Structural modeling suggested that this mRNA includes a stem-loop element that is similar to the anti-codon stem loop (ASL) structure of ThrRS cognate tRNA (tRNA^Thr^). We introduced random mutations within this element and found that almost every change from the normal sequence leads to reduced binding by ThrRS. Furthermore, point mutations at six key positions that abolish the predicted ASL-like structure showed a significant decrease in ThrRS binding with a decrease in RPC10 protein levels. Concomitantly, tRNA^Thr^ levels were reduced in the mutated strain. These data suggest a novel regulatory mechanism in which cellular tRNA levels are regulated through a mimicking element within an RNA polymerase III subunit in a manner that involves the tRNA cognate aaRS.

## 1. Introduction

Aminoacyl tRNA synthetases (aaRS) are a family of enzymes that recognize multiple cognate tRNAs and a single amino acid and charge the amino acid at the 3’ end of the tRNA with the expanse of an ATP [1]. Most cells have twenty different cytosolic aaRSs, each one responsible for ligating an amino acid to its cognate tRNAs. These enzymes are essential to cellular viability due to their central role in preparing the building blocks for ribosome activity, and any inaccuracy in their function is reflected in the protein produced. Multiple ‘identity elements’ in the target tRNAs are necessary for proper recognition by the aaRS. These elements are scattered through the entire RNA sequence and impose either a positive role (i.e., enhance binding to the correct aaRS) or a negative role (i.e., inhibit binding to an incorrect aaRS) [2]. For most aaRSs, a key determinant for recognition is the anticodon stem loop (ASL) region and in particular the anticodon itself. Mutations within this region were found in-vitro to reduce the correct binding to a proper aaRS and increase non-specific binding [3].

Interestingly, many aaRSs were found to have non-canonical functions in addition to their primary role in tRNA charging. These include cytokine activity, cellular signaling, or binding to DNA to regulate transcription [4,5]. It appears that the most abundant non-canonical role of aaRSs is in post-transcriptional regulation through direct binding to mRNA [6,7]. Studies in various organisms have indicated that binding may occur through structural elements that are similar to cognate tRNAs [6]. We recently surveyed the mRNA binding of most cytosolic yeast aaRSs and found a diversity in target mRNA binding and for some aaRSs a tendency to bind elements that are similar to cognate tRNAs [8]. This similarity can lead to a cross-talk between tRNA binding (and its charging) and mRNA binding (and its expression regulation) [6,9]. For example, binding of tRNA^Thr^ to ThrRS in *E.coli* leads to displacement of the target mRNA and relief of the inhibitory effect [10]. In yeast, overexpression of tRNA^His^ leads to reduced binding of HisRS to its target mRNA and increased translation [11]. 

Here, we tested the repertoire of mRNAs bound by ThrRS in *S. cerevisiae* cytosol. Interestingly, ThrRS shows an exceptional binding to an mRNA encoding a component of RNA polymerase III (RPC10). Point mutations revealed that binding occurs through a tRNA^Thr^ ASL-like element and has a role in the expression regulation of tRNA^Thr^ by RNA polymerase III. 

## 2. Results

### 2.1. mRNAs Bound by ThrRS

**Figure 1:**
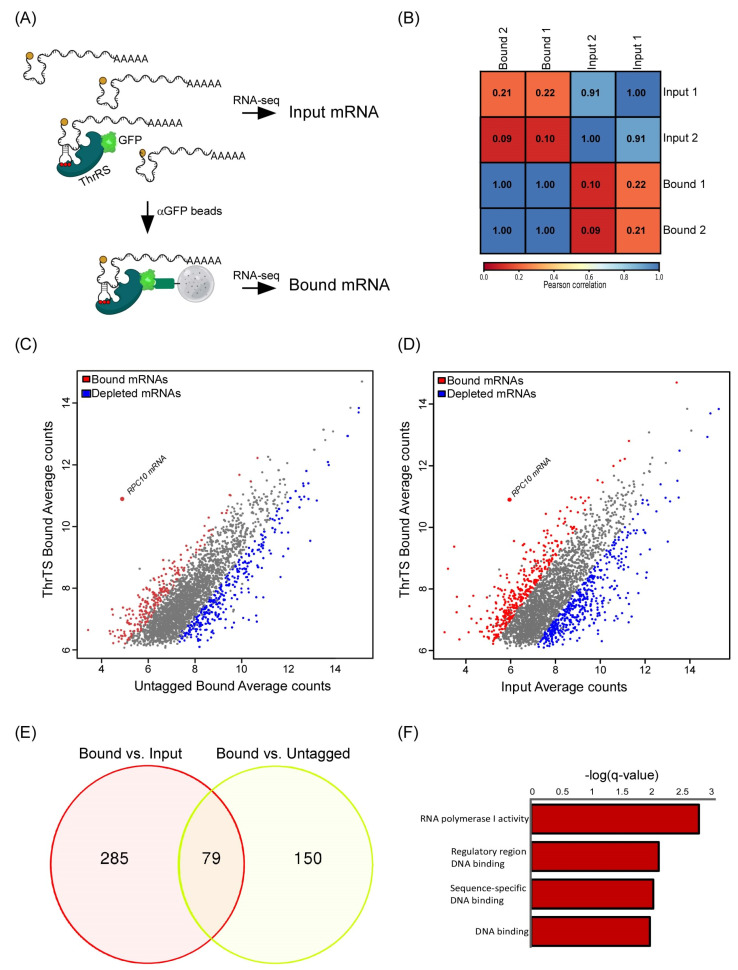
ThrRS mRNA binding analysis. (**A**) Scheme of the RIP -seq protocol. GFP-trap beads were used to isolate ThrRS-bound mRNAs from cell lysates of GFP-tagged ThrRS and untagged control strains. Bound and Input mRNA were subjected to RNA-seq. (**B**) Pearson correlation matrix between biological repeats and samples. (**C**) Scatterplot of mRNAs Bound signals in ThrRS-GFP vs. the untagged ThrRS control. Red dots indicate mRNAs significantly enriched (fold enrichment > 2 and adjusted *p*-value < 0.05), blue dots represent depleted mRNAs, and grey dots represent background mRNAs. (**D**) Scatterplot of mRNAs signals in the Bound vs. Input samples. Coloring as in (**C**). (**E**) Venn diagram was generated from significant Bound mRNAs compared to the Input (Red circle) and from significant Bound mRNAs compared to the Bound in the untagged control (yellow circle). (**F**) GO terms processes analysis was performed on the 79 bound mRNAs using SGD GO Term Finder Version 0.86.

To identify RNAs that ThrRS is associated with, we subjected GFP-tagged ThrRS (*THS1*) strain to RNA-binding protein immuno-purification (RIP) by anti-GFP magnetic beads. Isolated RNA (‘Bound’) from two independent biological repeats was subjected to high-throughput sequencing (RNA-seq) (Figure 1A) (Supplementary Table S1). RNA samples collected from the cell lysate before RIP (‘Input’) were also analyzed to account for differences in expression levels. Furthermore, the same RIP-seq procedure was applied to an untagged strain to account for non-specific interactions with the beads (Supplementary Table S1). A high correlation (Pearson correlation (*r_p_*) ~1) was observed between the two biological repeats of the Input and between the two of the Bound samples (Figure 1B). However, the correlation between the IP and the Input RNA data was much lower (*r_p_* < 0.22), suggesting that the reads are not merely reflections of mRNA expression. Furthermore, the correlation with the Bound signals from the untagged samples was similarly low (data not shown), which is consistent with low non-specific RNA binding. Overall, 364 mRNAs appeared enriched by more than two-fold in the Bound compared to the Input of the tagged strain, and 229 mRNAs were enriched in the Bound sample of ThrRS-GFP compared to the Bound of the untagged strain (Figure 1C,D). Seventy-nine mRNAs passed a double-sieve selection of being enriched compared to both controls (Figure 1E). Functional Gene Ontology (GO) term enrichment analysis (using SGD GO Term Finder Version 0.86) among the proteins encoded by these 79 mRNAs revealed enrichment of mRNAs encoding RNA polymerase subunits, in particular RNA polymerase I subunits and DNA binding proteins (GO:0001054 and GO:0000977) (Figure 1F) (Supplementary Table S2). 

### 2.2. RPC10 Is Bound by ThrRS through an Element Predicted to Form Cognate tRNA ASL

**Figure 2:**
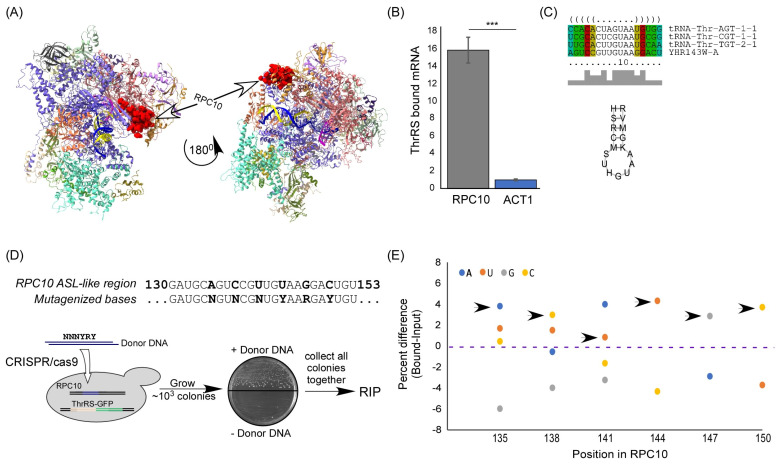
ThrRS binding to *RPC10* mRNA is mediated by an ASL mimic. (**A**) High-resolution Cryo EM structure of RNA polymerase III (PDB 5FJ8), RPC10 is in red, DNA strands are in yellow and blue, and RNA in magenta. (**B**) ThrRS-GFP-expressing yeast strains were subjected to RIP. Amounts of bound *RPC10* and *ACT1* mRNAs were quantified by RT-qPCR analysis and normalized to their expression levels (Input). The histogram presents the quantification of four independent biological repeats. *p*-value was calculated by the dependent samples’ one-tailed *t*-test. *** < 0.001. (**C**) tRNA^Thr^ and *RPC10* mRNA sequence and structure alignments. Three tRNA^Thr^ isoacceptors and *RPC10* (YHR143W-A) mRNA were subjected to multiple local and structure alignments of RNA molecules using LocARNA [16]. Alignment sequences are presented at the top and consensus stem-loop structure (letters designation according to IUPAC) at the bottom. (**D**) Scheme of CRISPR/Cas9 random mutagenesis of the anticodon-like region. Donor DNA (80 bases long) with randomized bases at the indicated positions was introduced into a strain expressing ThrRS-GFP. Double strand break at the RPC10 gene was induced by CRISPR/Cas9, and colonies grew only upon homologous recombination with the Donor DNA. The resulting recombinants, which contain varying sequences at RPC10 gene, were subjected to RIP. (**E**) RNA samples from the Input and Bound RIP analysis were subjected to RT followed by PCR of the RPC10 gene (with primers RPC10 seq F and RPC10 seq R)). Products were subjected to Sanger sequencing, and the fluorescent signals of each base in each mutated position were quantified. Graph presents the relative fluorescent differences (Bound minus Input) in each position for each base. Arrows point to the bases that are present in WT *RPC10*.

An mRNA that appeared significantly associated with ThrRS, far beyond all other mRNAs, is the mRNA encoding RPC10 (Figure 1C,D). RPC10 (YHR143W-A, ABC10α) is an essential, conserved, small subunit of all three RNA polymerases [12,13,14]. In RNA polymerase III, it is located between the two large catalytic subunits (Figure 2A). Mutations within this protein have a significant impact on tRNA expression and not other RNAs [15]. We validated RPC10 mRNA binding to ThrRS by RT-qPCR; *RPC10* mRNA is significantly enriched in the bound sample (~16 fold) compared to control mRNA (*ACT1*) (Figure 2B). 

We next wished to establish the mode of interaction between ThrRS and *RPC10* mRNA. LocARNA [16] was used for multiple sequence and structural alignments of *RPC10* mRNA and all tRNA^Thr^ isoacceptors. The analysis suggests a prominent anticodon stem loop (ASL)-like structure in *RPC10* mRNA at positions 135–151 that resembles the ASL of tRNA^Thr^ (Figure 2C). 

To examine the importance of this element for binding, in an unbiased manner, we introduced random mutations to this region (Figure 2D). This was achieved by utilizing a CRISPR/Cas9 approach that induces homologous recombination of a donor DNA with 6 random mutations, i.e., N, N, N, Y, R, and Y bases, in positions that correspond to A135, C138, T141, T144, G147, and C150 of *RPC10* coding region, respectively. Importantly, randomization was performed without affecting the encoded protein (all mutations are within wobble positions and do not drastically change the codon usage of the RNA). The use of CRIPSR/Cas9 mutagenesis protocol does not introduce any other change within this genomic locus, such as a selection marker [17]. Only a single colony grew when donor DNA was omitted (Figure 2D), indicating that cleavage efficiency is ~100%, and the majority of the colonies that grew upon donor DNA inclusion are products of recombination.

About 10^3^ recombinant yeast colonies (theoretically representing all 512 possible combinations of the six mutated sites) were grown to logarithmic phase in a rich medium and collected in a batch (parallel experiments in cells grown to stationary phase yielded similar results (not shown)). The sample was subjected to RIP with anti-GFP beads, and RNA was reverse transcribed and subjected to Sanger sequencing for *RPC10*. The fluorescent signal of each base is a proxy for its abundance within the analyzed population. A clear preference towards binding the WT transcript was apparent (Figure 2E): in almost all positions, the most efficiently bound base is the one that is present in the normal *RPC10* mRNA. The only exception is position 141, in which the normal base (U) is the second most bound. Overall, any change within the predicted ASL-like region leads to a reduced association. The enrichment towards the normal sequence is further appreciated considering the fact that the CRISPR/Cas9 scheme imposes a negative selection towards insertion of normal sequences. Thus, the predicted ASL-like sequence in *RPC10* is important for ThrRS binding.

### 2.3. Altered ThrRS Binding upon Elimination of the ASL-like Element

**Figure 3:**
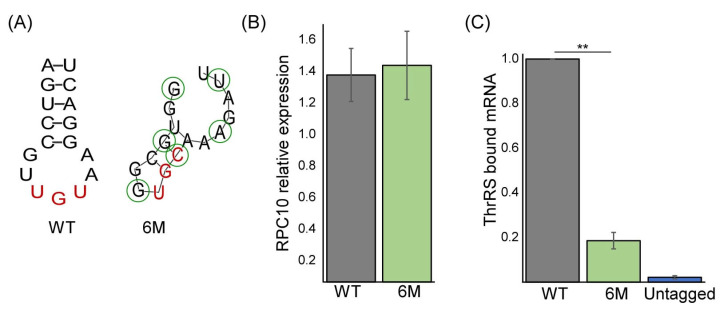
*RPC10* ASL-like structure is important for binding. (**A**) Structure prediction of the normal (WT) ASL-mimic in *RPC10* and the predicted the lowest binding efficiency mimic (6M). Green circles indicate mutated positions in 6M. (**B**) Steady-state levels of wildtype (WT) and mutant (6M) *RPC10* mRNA were quantified by RT-qPCR analysis from three independent biological repeats, each with three technical repeats, normalized to *ACT1* mRNA levels. (**C**) ThrRS-GFP expressing yeast strains, endogenously co-expressing either WT or 6M *RPC10* mRNA and an untagged control, were subjected to RIP. Amounts of bound *RPC10* mRNAs were quantified by RT-qPCR analysis and normalized to their expression levels (Input levels). The histogram presents the quantification of five independent biological repeats. *p*-value was calculated by the dependent samples’ one-tailed *t*-test. ** < 0.01.

To specifically validate the importance of the binding element, we generated a strain expressing *RPC10* with six specific mutations in this region: A135G, C138G, U141G, U144C, G147A, and C150U (Figure 3A)(*RPC10-6M*). This combination was selected based on the RIP followed by Sanger sequencing results (Figure 2E) to represent the least associated *RPC10* mRNA variant. Structural prediction suggests a significant change in the structure of the region (Figure 3A). Expression analysis revealed similar levels of the *RPC10* transcript from the WT or *RPC10-6M* cells (Figure 3B). Nevertheless, RIP followed by RT-qPCR revealed a five-fold decrease in ThrRS association with the mutated transcript compared to the WT transcript (Figure 3C). Notably, binding of the 6M variant was higher than it was to the untagged control ThrRS, indicating that additional regions of *RPC10* may also be involved in binding. This targeted mutagenesis further substantiates the notion that the predicted ASL within *RPC10* is important for ThrRS binding.

### 2.4. The Predicted ASL-Mimic Is Important for RPC10 Translation 

**Figure 4:**
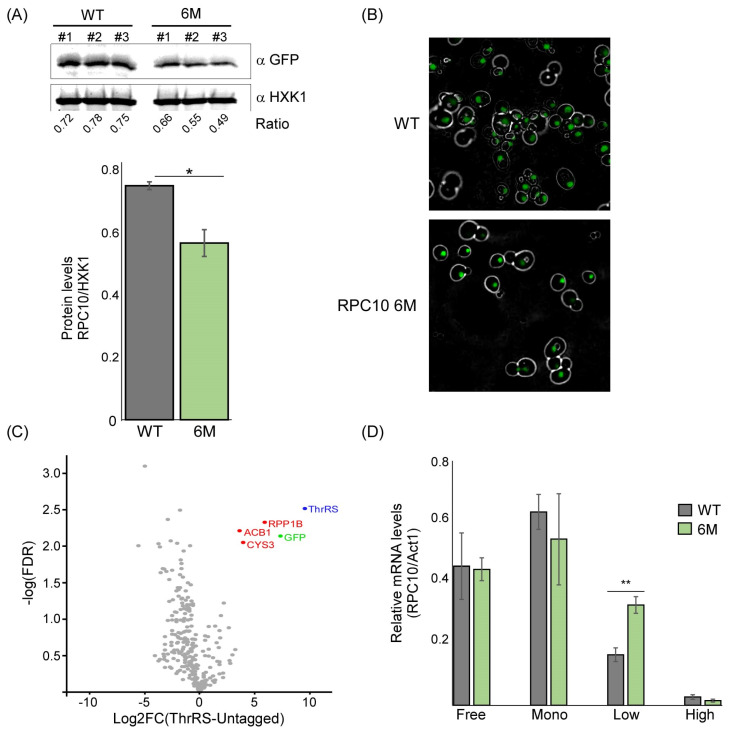
ThrRS impact on *RPC10* expression. (**A**) Western analysis for RPC10-GFP expressed either from the normal transcript or the 6M mutant. The ratio is the quantification ratio between the ThrRS-GFP and Hxk1 signals of three independent biological repeats. Histogram presents the quantification average, error bars are SEM, and *p*-value was calculated by the dependent samples’ one-tailed *t*-test. * < 0.05 (**B**) GFP localization of RPC10 expressed from WT or 6M mutant mRNA. Representative images obtained by spinning disk confocal microscope. (**C**) ThrRS-GFP cells were subjected to Co-IP, and purified proteins were subjected to LC-MS/MS. Results are from two independent biological repeats for each protein, and the fold-change of its label-free quantitation (LFQ) intensity compared to untagged control was calculated. Scatterplot indicates the log_2_ fold-change (compared to untagged control) for each protein defined by MS/MS vs. the significance (as −log10 of the FDR). Red-marked dots indicate proteins that are defined as significant (FDR < 0.05 and log_2_ fold-change > 2). (**D**) Sucrose gradient fractions of samples from WT or *RPC10 6M* cells were analyzed by RT-qPCR for the indicated transcripts. ‘Free’ fraction includes all mRNAs up to monosome size, ‘Mono’ represents mRNAs with a single ribosome, ‘Low’ represents mRNAs with 2–4 ribosomes, and ‘High’ represents polysomes with 5 or more ribosomes. The histogram presents the quantification of three independent biological repeats. *p*-value was calculated by the dependent samples’ one-tailed *t*-test. ** < 0.01.

RNA binding proteins exert a post-transcriptional impact on their targets primarily through affecting mRNA stability and translation. ThrRS was previously implicated in mRNA decay [18]. Nevertheless, we did not observe a strong impact on *RPC10* transcript levels upon reduced ThrRS binding (Figure 3B). We therefore focused on the possible impact on translation. Western analysis of a GFP-tagged RPC10-6M revealed a significant decrease in protein levels compared to the WT (Figure 4A). This decrease is not reflected in the localization of the protein, as RPC10 expressed from the mutated mRNA is still fully localized to the nucleus (Figure 4B). Importantly, these six mutations do not affect the encoded protein as they fall within wobble positions. We used stAIcalc, a tRNA Adaptation Index (tAI) calculator based on species-specific weights [19], to calculate the effect of these mutations on translation efficiency and did not identify a dramatic change in the tAI (from 0.3161 in the WT to 0.3015 in the 6M mRNA). 

We hypothesized that the changes in protein levels are due to translation regulation specific to *RPC10* mRNA, mediated by ThrRS binding. To uncover such regulation, we searched for protein partners of ThrRS that may be involved in translation regulation. GFP-tagged ThrRS was immunoprecipitated, and its associated proteins were identified by LC-MS/MS (Supplementary Table S3). Analysis of the LC-MS/MS results revealed that ThrRS (Ths1p) is highly enriched by 629-fold compared to the untagged control (Figure 4B), as well as its GFP-fused moiety. This result suggests the high efficiency and specificity of the isolation protocol. Overall, three proteins were detected to significantly interact with ThrRS (log_2_ fold-change > 2 and FDR < 0.05). Interestingly, two of them are proteins involved in metabolic pathways (ACB1 and CYS3). Recently, we revealed that aaRS interacts with mRNAs that encode proteins involved in amino acid metabolism, suggesting that aaRS can post-transcriptionally regulate amino acid biosynthesis [8]. Theprotein–protein interaction of ThrRS with CYS3, an enzyme involved in cysteine biosynthesis, suggests that ThrRS may also post-translationally regulate amino acids biosynthesis. 

Remarkably, ThrRS’s most significant protein partner is RPP1B (Figure 4B). RPP1B is part of a heteromeric complex that associates with the ribosomal stalk (P1β). It is known to interact with translational elongation factors [20,21], therefore suggesting a role for ThrRS in the elongation phase of translation. To examine that, polysome profiling through sucrose gradients was performed on both strains (WT and 6M). Consistently with a previous report [22], *RPC10* appeared associated with 2–4 ribosomes in the WT strain (i.e., low polysomal fraction) (Figure 4C). Importantly, RPC10 mRNA with the mutated ASL-like element (*RPC10-6M*) is observed at higher levels in this fraction. This increase may emerge from the shift in the transcript from the monosome fraction, which shows lower levels of RPC10-6M transcript. 

### 2.5. Mutations within the Predicted ASL-like Site Lead to an Impact on tRNA^Thr^ Expression

**Figure 5:**
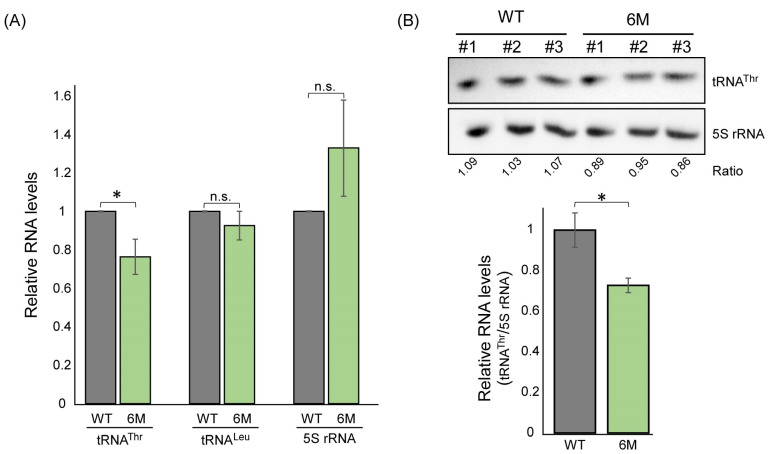
RPC10 impact on tRNA^Thr^ expression. (**A**) Amounts of tRNA^Thr(AGU)^, tRNA^Leu(CAA),^ and 5S rRNA were quantified in the indicated strains by RT-qPCR analysis and normalized to *ACT1* expression levels. The histogram presents the quantification of five independent biological repeats. (**B**) RNA samples (2.5 μg) from the indicated strains (three biological replicates each) were resolved on 10% acrylamide/8 M Urea gel and subjected to northern analysis with Biotin-labeled probes followed by detection with HRP-conjugated streptavidin and an ECL reaction. ’Ratio’ is the quantification ratio between the tRNA and 5S signals in each lane. Histogram presents the 5S-normalized tRNA^Thr^ quantification. *p*-value was calculated by the dependent samples’ one-tailed *t*-test. * < 0.05.

RPC10 is a component of RNA polymerase III with a role in tRNA expression. We therefore tested whether regulation of its translation by ThrRS has an impact on the expression of tRNA genes. RT-qPCR or northern analyses of tRNA^Thr (AGU)^ levels in WT and RPC10-6M mutants revealed a decrease in tRNA^Thr^ levels. However, other tRNAs (tRNA^Leu^) or 5S ribosomal RNA are not significantly changed (Figure 5). This suggests a specific impact of decreased RPC10 levels on tRNA^Thr^ transcription. 

## 3. Discussion

### 3.1. The Importance of the Predicted ASL-like for Translation Regulation of RPC10

In this work, we present indications for the presence of an ASL-like element within *RPC10* mRNA, with a role in regulating its translation into protein. Introducing random mutations within this element, which are expected to deform its ASL structure without altering the encoded protein, revealed that practically every change in the predicted ASL-like element reduced the binding of ThrRS to *RPC10* mRNA. This result reveals the importance of this particular ASL-like sequence for binding. A role of the ASL of yeast tRNA^Thr^ in recognition by ThrRS was described previously by in-vitro assays [23]. Notably, mutating position 36 of the tRNA led to a significant decrease in tRNA acylation. This position aligns with position 144 of RPC10, which appears to be important for in-vivo binding (Figure 2). However, our mutagenesis scheme within RPC10 mRNA was not exhaustive, as we did not want to affect the encoded RPC10 protein; therefore, detailed alignment to all in-vitro described [23] tRNA^Thr^ ASL identity elements is not provided. On the other hand, as we did not affect the encoded protein by mutagenesis, we were able to test the impact of the ASL-like element on RPC10 translation. Our data reveal that steady-state protein levels of RPC10 decrease upon disruption of this ASL-like element. This suggests a translation activation role for ThrRS binding. This is different from *E.coli* ThrRS, which was found to repress translation of a target mRNA (actually its own mRNA) through binding a tRNA-like element within its 5′ UTR element [24,25]. Yet, this is consistent with the ThrRS mouse homolog, which was also found to increase its target mRNAs translation through binding to a cap-binding protein (eIF4E2). Nevertheless, the yeast ThrRS did not bind eIF4E [26], suggesting a different mechanism of activation. 

**Figure 6:**
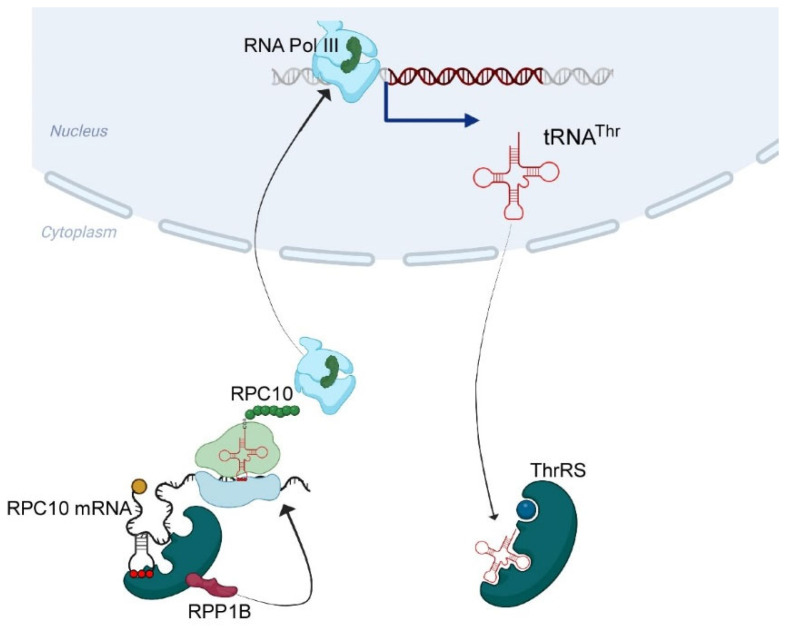
Suggested model for tRNA^Thr^ expression regulation through ThrRS. Our data reveal that ThrRS binds *RPC10* mRNA in an anticodon-like structure. We propose that bound ThrRS recruits RPP1B to locally mediate synthesis of RPC10 protein. RPC10 protein then binds RNA polymerase III and regulates transcription of tRNA^Thr^.

Our data show an increase in RPC10 protein level upon mutations within the ASL-like element. In parallel, the ribosomal association of the mutated transcript is increased (Figure 4). These changes are most consistent with the impact on translation elongation, in which the increased number of ribosomes translate more slowly on the mutated transcript and thereby generate a lower amount of protein compared to the normal transcript. It is possible that ThrRS binding to mRNA within the coding region leads to an impact on ribosome transit. Upon mutation, ThrRS association with RPC10 mRNA decreases, resulting in an increase in *RPC10* mRNA ribosomal association and a reduction in RPC10 protein levels, consistent with reduced ribosome transit time along the *RPC10*-coding region (Figure 4C). Analysis of ThrRS-bound proteome revealed that it preferentially interacts with the ribosomal protein P1 beta (RPP1B) (Figure 4C). This protein is a component of the ribosomal stalk, which facilitate the interaction of translational elongation factors with the ribosome. The stalk acidic proteins (P1 and P2) are ribosomal components that are found free in the cytoplasm [27]. During protein synthesis, the ribosome-bound P1 and P2 are exchanged with the free cytosolic subunits, and it has been hypothesized that this can regulate ribosome activity (i.e., translation elongation rate) [20,28]. These data suggest that ThrRS bring RPP1B in proximity to *RPC10* mRNA to locally increase ribosomes elongation rate, which leads to a reduced number of ribosomes on the mRNA (Figure 6).

### 3.2. Regulation of Expression by tRNA Mimicry

The data presented here suggest a mechanism that phenocopy tRNA features to post-transcriptionally regulate mRNA expression. Structural elements that resemble anticodon moieties of cognate tRNAs are likely to be involved. The functional significance of this mimic is supported both by random mutagenesis, in which it appears that almost every deviation from the WT, structural ASL-like motif has a lower association with ThrRS, and by the six point-mutations that specifically altered the predicted ASL-like element and led to a clear reduction in ThrRS association. 

Binding through RNA structural mimicry was revealed years ago for the *E. coli* HisRS and ThrRS [10,24,29,30]. In addition, tRNA mimicry was found to be important in *S. cerevisiae* AspRS self-association [31,32]. RNA mimicry is a broad phenomenon and is not restricted to tRNAs [6]. A well-established case is the regulation of *E. coli* ribosomal protein expression. *E. coli* ribosomal RNA operators contain structural elements that resemble the binding sites of these proteins within rRNA (rRNA mimicry) [33,34]. When ribosomal proteins are in excess, they bind these rRNA mimics within their operators and repress their own translation. Thus, binding through RNA mimicry might be a general property of RBPs that bind different types of RNA. Our results expand this knowledge by providing another example of such mimic in a eukaryotic mRNA. 

### 3.3. Regulation of tRNA Expression by ThrRS 

We present here an indication of a novel regulatory mechanism, in which aaRS acts as a tRNA expression regulator by affecting RNA polymerase III transcription activity (Figure 6). Recently, a cytosolic co-translational assembly mechanism was suggested for RNA polymerase III, based on studies on another RNA polymerase III small subunit [35]. It is yet to be determined whether RPC10 is also involved in co-translational assembly; nevertheless, in such a case, the ThrRS-mediated translation of RPC10 can directly affect RNA polymerase III assembly and transcriptional activity. We therefore propose that when tRNA^Thr^ levels are lower than necessary, a free ThrRS binds *RPC10* mRNA and through RPP1B affects ribosomes movement to increase RPC10 protein levels. Increased *RPC10* translation supports RNA polymerase III cytosolic assembly and activates transcription of tRNA^Thr^ to restore its proper levels (Figure 6). Of note, previous mutagenesis analysis identified mutants of RPC10 that affect only RNA polymerase III and not polymerase II targets [15]. Moreover, RPC10 interacts with the TFIIIC complex, a general transcription factor that is required for the assembly of RNA polymerase III [36]. Thus, although RPC10 is a component of the RNA polymerase I and II complexes, it may impose an RNA polymerase III-specific function. 

In sum, our data is consistent with a growing body of work that reveal the presence of tRNA-like sequences within mRNAs, and their importance for RBP binding. Furthermore, we suggest that these elements allow coordination between the expression of a target tRNA and the tRNA-mimic-containing mRNA. This underscores the importance of RNA mimics for cellular regulation.

## 4. Materials and Methods

### 4.1. Yeast Strains, Growth Conditions, and Plasmids

The parental yeast strain for all studies is BY4741 (Mat a, *his3∆1, leu2∆0, met15∆0, ura3∆0*), an S288C-derivative laboratory strain (Table 1). GFP-fusion for ThrRS (THS1) is from the GFP C terminal collection and shows cytosolic expression of the fusion protein (kindly provided by Prof. Maya Schuldiner). The addition of the GFP moiety to THS1 did not affect its transcript level, as measured by RNA-seq of tagged vs. untagged strains (data not shown). Cells were usually grown in liquid or on plates of YPD (1% Yeast extract 2% Bacto peptone, 2% glucose) or YPG (1% Yeast extract 2% Bacto peptone, 2% galactose) at 30 °C. Plasmids for CRISPR/Cas9 were derived from bRA66 (pA1067), into which a double-stranded DNA was inserted into the BplI site, resulting in an expression of a gRNA that induces a double-strand break at position 143 of RPC10 gene (pA1194, Table 2).

### 4.2. RBP ImmunoPrecipitation (RIP) and RNA-seq Analyses

RIP was essentially as described in [11]. Briefly, strains were grown in YPD to mid-logarithmic phase and subjected to cross-linking by the addition of formaldehyde (0.05% final concentration) for 10 min at room temp. Cross-linking was terminated with 0.125 M glycine for 3 min, and cells were lysed using a bead beater. Lysate was cleared by centrifugation at 10,600× *g* for 10 min at 4 °C. Lysates were loaded on GFP-Trap_A (ChromoTek Catalog gta) and rotated at 4 °C for 2 h. Samples were washed four times and eluted by 0.2 M Glycine buffer (pH 2.5). Cross-linking was reversed by heating at 65 °C for 2 h in reverse cross-linking buffer, and RNA was precipitated after phenol:choloroform extraction.

RNA samples were subjected to RNA-seq at the Technion Genome Center. Libraries from the Input and Bound RIP samples were prepared using a TruSeq RNA Library Prep Kit v2 (Illumina, CA, USA) according to the manufacturer’s instructions. All samples were sequenced on Illumina platform, yielding 20 to 30 million reads per sample. Reads were mapped to the S288c *S. cerevisiae* version R64–2-1 genome using RNA STAR version 2.6.0b-2. Only uniquely mapped reads were counted for genes using HTSeq-count package version 0.6.1.

### 4.3. CRISPR/Cas9-Mediated Recombination

Homologous recombination by CRISPR/Cas9 was performed as described in [17]. Cells in the early exponential phase (50 mL) were collected by centrifugation at 4000 RPM for 4 min at 25 °C and washed once with sterile water and pelleted at 4000 RPM for 4 min at 25 °C. Pellets were resuspended in 0.4 mL of 0.1 M LiAc, and 100 µL per transformation reaction was used. Each 100 µL fraction was pelleted and suspended in 40 µL sterile water, 36 µL 1 M LiAc, 25 µL ssDNA (2 mg/mL), and 4 mL (2 μg) plasmid (Table 2) and 30 µL donor DNA (100 mM stock)(Table 3) and 240 µL 50% PEG. Samples were incubated for 30 min at 30 °C followed by 15 min at 42 °C. Transformation mixtures were plated on YPG supplemented with 100 μg/mL Hygromycin B plates. Positive colonies were collected after 2 days and replated on a selection medium for verification.

### 4.4. Polysome Analysis

Polysome analysis was essentially as described in [37]. Briefly, 50 mL of cells grown in YPD to the mid-logarithmic stage were harvested and lysed by a bead beater in 0.4 mL of lysis buffer (20 mM Tris–HCl at pH 7.4, 140 mM KCl, 1.5 mM MgCl_2_, 0.5 mM dithiothreitol, 100 μg/mL cycloheximide, 0.24 U/μL RiboLock RNase Inhibitor (Thermo Scientific, Waltham, MA, USA), 1% Triton X-100). The lysate was cleared and centrifuged for 15 min at 9000× *g* at 4 °C, and the supernatant was loaded onto a 12 mL 10–50% linear sucrose gradient. Gradients were centrifuged in a SW41 rotor (Beckman-Coulter, Brea, CA, USA) at 35,000 rpm for 160 min, and the entire gradient was fractionated into four fractions. RNA was extracted from each fraction by the addition of an equal volume of 8 M guanidinium HCl and two volumes of 100% ethanol, incubated overnight at −20 °C, and centrifuged at 13,000 rpm for 30 min. Pellets were washed with 80% cold ethanol, and 1 µg of RNA sample was subjected to RT-qPCR with the indicated oligos (Table 3).

### 4.5. tRNA Quantification by RT-qPCR

tRNA RT-qPCR was performed as described in [38]. Briefly, tRNA was reverse-transcribed using a RevertAid First Strand cDNA Synthesis Kit (Thermo Fisher Scientific, Waltham, MA, USA). The reaction temperature was elevated to 60 °C, and reverse-transcription elongation was extended to 30 min to efficiently overcome tRNA modification and secondary structure. tRNA-specific levels were determined in a 20 μL reaction volume in triplicate with a Power SYBR Green PCR Master Mix^®^ (Applied Biosystems, Waltham, MA, USA) following the manufacturer’s instructions using primers for the indicated tRNA as described in [38] (Table 3). Results were analyzed with Applied Biosystems 7500 Real-Time PCR Softwarev2.0.6. Fold change was calculated using 2^−(ΔCt).^

### 4.6. Protein Co-Immunoprecipitation

A strain expressing GFP-tagged ThrRS (YA1545) and an untagged control (YA1) (250 mL) were grown in YPD to mid-logarithmic phase and subjected to cross-linking by the addition of formaldehyde (1% final concentration) for 10 min at room temp. Cross-linking was terminated with 0.125 M glycine for 3 min, and cells were lysed using Bead Beater in Buffer B (20 mM Tris-HCl (pH 7.5), 140 mM NaCl, 0.1% NP40, 0.5 mM EDTA, 1 mM DTT, 2 mM PMSF, 10 μg/mL Leupeptin, 14 μg/mL Pepstatin, and 0.02 U/×L RQ1 RNase-free DNase (Promega, Madison WI, USA)). The lysate was cleared by centrifugation at 10,600ρ *g* for 10 min at 4 °C. Lysates were loaded on GFP-Trap_MB (ChromoTek, gtmb, Munchen Germany) and rotated at 4 °C for 2 h. Samples were washed four times in Buffer C (20 mM Tris-HCl (pH 7.5), 0.5 M NaCl, 0.5% NP40, 0.5 mM EDTA, 0.5 mM DTT, 0.01 U/μL RiboLock RNase Inhibitor (Thermo Fisher Scientific, Waltham MA, USA)). Then, samples were washed three times in PBS and eluted with 0.2 M Glycine buffer (pH 2.5).

### 4.7. Mass Spectrometry (MS/MS) Analysis

Samples from protein co-immunoprecipitation (two biological repeats) were digested by trypsin, analyzed by LC-MS/MS on Q-Exactive (Thermo Fisher Scientific, Waltham, MA, USA), and identified by MaxQuant_2.0.1.0 software against the *Saccharomyces cerevisiae* proteome database. Enriched proteins were calculated as the ratio between the LFQ Intensity of the protein in the ThrRS-tagged GFP and untagged ThrRS samples. Enrichment of proteins for the GFP tagged ThrRS against untagged was set to fold enrichment >2, adjusted *p*-value < 0.05 (Supplementary Table S3).

**Table 1:** Yeast strains.

Lab Code	Strain Name	Description	Genotype	Source
YA1	BY4741	Wild type	*Mat a, his3∆1, leu2∆0, met15∆0,ura3∆0*	[39]
YA1545	THS1-GFP	THS1 (YIL078W) C-terminally tagged with GFP, in BY4741	*MATa, leu2Δ0, met15Δ0, ura3Δ0, THS1: GFP (S65T)-HIS3*	[40]
YA1595	THS1-GFP RPC10-6M	THS1 (YIL078W) C-terminally tagged with GFP, BY4741. RPC10 gene was mutated (AGTCCGTTGTAAGGAC to GGTGCGGTGCAAAGAT)	*MATa, leu2Δ0, met15Δ0, ura3Δ0, THS1: GFP (S65T)-HIS3, rpc10-6m*	This study
YA1619	GFP-RPC10	N terminally GFP-tagged RPC10 (YHR143W-A), BY4741	*MATa, leu2Δ0, met15Δ0, ura3Δ0, GFP(S65T)-HIS3: RPC10*	[41]
YA1620	GFP-RPC10 6M	N terminally GFP-tagged RPC10 (YHR143W-A), BY4741. RPC10 gene was mutated (AGTCCGTTGTAAGGAC to GGTGCGGTGCAAAGAT)	*MATa, leu2Δ0, met15Δ0, ura3Δ0, GFP(S65T)-HIS3: RPC10*	This study

**Table 2:** Plasmids.

Lab Code	Plasmid Name	Description	Source
pA1067	bRA66	(Empty Backbone) GAL1 driven Cas9 Addgene #100952	[42]
pA1194	RPC10 + 143 gRNA	bRA66 with RPC10 +143 (AACTGATGCAGTCCGTTGTA) targeting gRNA cloned into BplI sites	This study

**Table 3:** Primers and Oligos.

**gRNA Oligos**	
**Primer Name**	**Primer sequence**
RPC10 gRNA-143 F	AACTGATGCAGTCCGTTGTAGTTTT
RPC10 gRNA-143 R	TACAACGGACTGCATCAGTTGATCA
**Donor DNA Oligos**	
**Primer Name**	**Primer sequence**
RPC10 Random Donor DNA F	TAGTAAATTATCTTTATCCAGAACTGATGC**N**GT**N**CG**N**TG**Y**AA**R**GA**Y**TGTGGTCATAGAATCCTGTTGAAGGCTAGGACTA
RPC10 Random Donor DNA R	TAGTCCTAGCCTTCAACAGGATTCTATGACCACA**R**TC**Y**TT**R**CA**N**CG**N**AC**N**GCATCAGTTCTGGATAAAGATAATTTACTA
RPC10 6M Donor DNA F	TAGTAAATTATCTTTATCCAGAACTGATGCgGTgCGgTGcAAaGAtTGTGGTCATAGAATCCTGTTGAAGGCTAGGACTA
RPC10 6M Donor DNA R	TAGTCCTAGCCTTCAACAGGATTCTATGACCACAaTCtTTgCAcCGcACcGCATCAGTTCTGGATAAAGATAATTTACTA
**qPCR Oligos**	
**Primer Name**	**Primer sequence**
RPC10 seq F	ATGTCTCGCGAAGGGTTC
RPC10 seq R	TCAAATTGAACCAATCTCTTAGTCC
**RT-qPCR Oligos**	
**Primer Name**	**Primer sequence**
RPC10 F	CGCGAAGGGTTCCAGATT
RPC10 R	CTTCAAAGTTGCCGTTCTAGC
Act1 F	GATTCTGAGGTTGCTGCTTTG
Act1 R	ACCGACGATAGATGGGAAGA
tRNA-Thr-AGT F	CAAGTTGGTAAGGCGCCAC
tRNA-Thr-AGT R	TCGGATTTGAACCGATGATCTCC
tRNA-Leu-CAA F	TTTGGCCGAGCGGTCTAAGG
tRNA-Leu-CAA R	TGCATCTTACGATACCTGAGCTT
5S rRNA F	GCGGCCATATCTACCAGAAA
5S rRNA R	GTATGGTCACCCACTACACTAC
**Northern probes**	
tRNAThr biotin	/5BiosG/GGATTTGAACCGATGATCT
5S biotin	/5BiosG/TGGTAGATATGGCCGCAACC

## Data Availability

The authors confirm that the data supporting the findings of this study are available within the article and its supplementary materials.

## Supplementary Materials

The following supporting information can be downloaded at https://www.mdpi.com/article/10.3390/genes14020462/s1: Table S1: ThrRS-bound mRNAs; Table S2: ThrRS-bound GO terms; Table S3: ThrRS-bound proteome.
